# Supplementary material for: Cerebrospinal Fluid α-Synuclein Species in Cognitive and Movements Disorders
Source: Brain Sci. 2021 Jan 17;11(1):119. doi: 10.3390/brainsci11010119 (PMC7830324; doi:10.3390/brainsci11010119)
Supplement: Supplementary file 1 [file brainsci-11-00119-s001.pdf]

	cut-off	AUC (SD)	Sig.	Sens.	Spec.	LR	YI
All patients							
t-α-syn	≤865pg/ml	0.687	0.034	86%	59%	2.1	0.45
Patients with no blood contamination							
t-a-syn	≤865pg/ml	0.828	0.002	94%	69%	3.1	0.63
pS129-α-syn/t-α-syn	≥0.122	0.749	0.021	71%	77%	3.1	0.48

Sup. Table 1. ROC curve analysis of a-syn for the discrimination of synucleinopathies from tauopathies. AUC: area under the curve; Sens: sensitivity; spec: specificity; LR: Likelihood ratio; YI: Youden's index

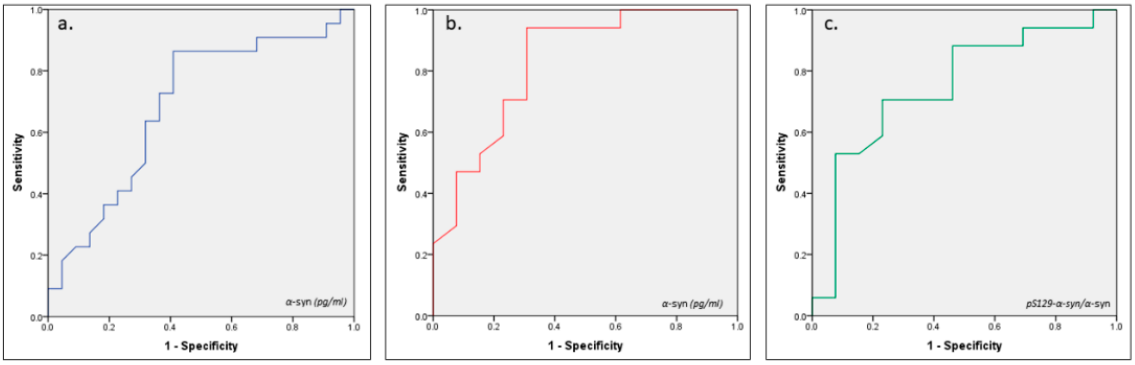

Sup. Figure 1. Receiver Operating Characteristic (ROC) curve analyses of the diagnostic accuracy of various α-syn species for the differential diagnosis of synucleinopathies from tauopathies. a: CSF α-syn in all patients; b: CSF α-syn in patients after excluding CSF samples with blood contamination; c: CSF pS129-α-syn/α-syn ratio in patients after excluding CSF samples with blood contamination.

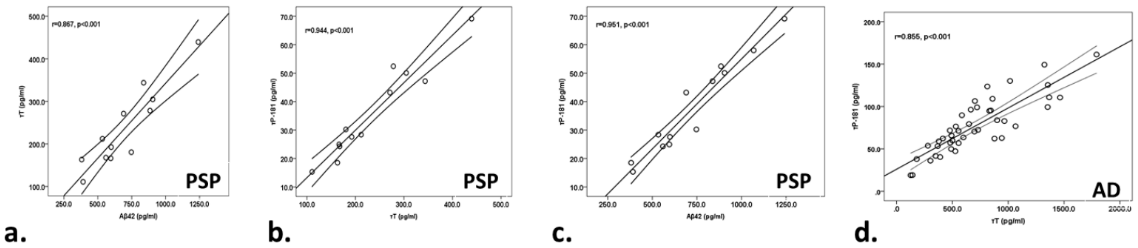

Sup. Figure 2 Scatterplots of Spearman correlations between CSF biomarkers.
